# Patient characteristics and outcome in three different working models of home-based rehabilitation: a longitudinal observational study in primary health care in Norway

**DOI:** 10.1186/s12913-021-06914-2

**Published:** 2021-08-28

**Authors:** Ingebrigt Meisingset, Joakim Bjerke, Kristin Taraldsen, Mari Gunnes, Sylvi Sand, Anne E. Hansen, Gard Myhre, Kari Anne I. Evensen

**Affiliations:** 1 Department of Public Health and Nursing, NTNU, Trondheim, Norway; 2 Unit for Physiotherapy Services, Trondheim Municipality, Trondheim, Norway; 3 Department of Neuromedicine and Movement Science, Norwegian University of Science and Technology (NTNU), Trondheim, Norway; 4 Department of Clinical and Molecular Medicine, NTNU, Trondheim, Norway; 5 Department of Physiotherapy, Oslo Metropolitan University, Oslo, Norway

**Keywords:** Functional decline, Prevention, Rehabilitation, Health-related quality of life, Home-based physiotherapy, Mobility, Older adults, Physical function, Primary health care, Reablement

## Abstract

**Background:**

The organisation of health care services for older adults varies within and between countries. In Norway, primary care physiotherapy services offer home-based rehabilitation to older adults. The aim of this study was to compare patients’ characteristics and treatment outcomes in three working models of home-based rehabilitation.

**Methods:**

Patients referred to home-based rehabilitation in a large municipality in Norway were invited to participate in this prospective observational study. The three working models, early intervention, reablement and regular physiotherapy, were organised according to the patients’ function and degree of independence. The older adults (≥ 65 years) were allocated to the different models by either a multidisciplinary group of health care personnel or by direct referral. Patients’ demographic and clinical characteristics, including physical function (Patient-specific functional scale, PSFS), physical performance (Short Physical Performance Battery, SPPB) and health-related quality of life (EQ-5D) were registered at baseline and follow-up (maximum 6 months after baseline). One-way ANOVA was used to analyse group differences in clinical characteristics and paired t-tests to analyse changes from baseline to follow-up.

**Results:**

In total, 603 and 402 patients (median (interquartile range) age: 84 (77–88) years) completed baseline and follow-up assessments, respectively. Patients in all three working models had an increased risk for functional decline. Patients receiving early intervention (*n* = 62) had significantly (*p* < 0.001) better physical performance and health-related quality of life (SPPB mean 7.9, SD 2.7; EQ-5D:mean 0.59, SD 0.19), than patients receiving reablement (*n* = 132) (SPPB: mean 5.5, SD 2.6; EQ-5D: mean 0.50, SD 0.15) and regular physiotherapy (*n* = 409) (SPPB: mean 5.6, SD 2.8; EQ-5D: mean 0.41, SD 0.22). At follow-up, the three working models showed significantly improvements in physical function (PSFS: mean change (95 % CI): 2.5 (1.9 to 3.2); 1.8 (0.5 to 3.1); 1.7 (0.8 to 2.6), for regular physiotherapy, reablement, and early intervention, respectively). Patients receiving regular physiotherapy and reablement also significantly improved physical performance and health-related quality of life.

**Conclusions:**

While older adults receiving reablement and regular physiotherapy showed similar patient characteristics and treatment outcomes, early intervention identified older patients at risk of functional decline at an earlier stage. These results are relevant for policy makers when designing and improving prevention and rehabilitation strategies in primary health care.

## Background

Changes in demography nationally and globally, with a higher proportion of older adults and increased longevity [1], along with limited resources in the health care system, emphasises the importance of early prevention and postponing the need for home-based services and residential care. Home-based interventions are implemented in several countries to enable older adults to live longer in their homes. There is, however, limited knowledge on how to best organise the home-based interventions to meet the challenges of an aging population, and to which extent the services are targeted to the individual patient’s functional status [2–4].

Home-based rehabilitation services, also called reablement or restorative care, are differently organised and conceptualised within and between countries [3–9], making comparisons across studies challenging. The Norwegian primary health care system offers home-based rehabilitation to older adults at risk of, or already experiencing functional decline. A recent study found substantial variation across municipalities in Norway in the organisation, content, and target group for reablement [6], which may influence the quality of care and the intervention outcome. Langeland et al. investigated the effect of reablement in participants experiencing a recent functional decline in Norway in a non-randomised study including 47 municipalities. They found that the treatment outcome, in terms of activity performance and satisfaction of performance, was similar across municipalities in Norway with different organisation of reablement [3]. However, they did not report the functional status of the participants across different organisational models, which could have indicated different target groups across the municipalities in terms of functional status. Further, another recent study from Norway reported the functional status, measured by the Short Physical Performance battery (SPPB), in patients admitted to nursing homes [10]. The functional status of these patients was somewhat lower, but surprisingly similar, to the functional status of those receiving reablement in a home-setting in the study of Langeland et al. [3, 10]. These findings indicate that reablement is initiated when the functional status has deteriorated substantially, and thus, the prevention potential of reablement services may be limited.

Physiotherapists (PTs) play a central role in preventing functional decline and maintaining independence in activities of daily life. In a municipality in Norway, attempts have been made to identify older adults at risk for functional decline at an earlier stage, a so-called “early intervention” model. This model consists of multidisciplinary rehabilitation, delivered by PTs, health and welfare officers, and occupational therapists. This is in contrast to a reablement model where PTs prescribe home exercise plans, which is followed up by the home-based care services on a regular basis [6], or the traditional approach with individually home-based physiotherapy, in the present study called regular physiotherapy. However, no studies have reported on patient characteristics and thus the target group of these organisational models of home-based rehabilitation. There is thus a lack of knowledge on how different organisational models and structures of home-based rehabilitation reflects different functional status, other important health aspects, and the intervention outcome of older adults receiving home-based rehabilitation services.

The overall aim of this study was therefore to compare (1) demographics, functional status and clinical characteristics of older adults receiving different models of home-based rehabilitation in primary health care. Further, we compared (2) changes in physical performance, physical function, and health-related quality of life from baseline to follow-up.

## Methods

### Design and setting

Through the Research program for Physiotherapy in Primary Health Care, the FYSIOPRIM, a set of standardised methods and tools have been developed, enabling studies of clinical courses for patients receiving primary care physiotherapy [11]. The present study is a prospective longitudinal observational study of older adults receiving home-based rehabilitation in Trondheim Municipality, which is located in the middle part of Norway. Trondheim has around 205 000 inhabitants and is the third largest municipality in Norway.

**Fig. 1:**
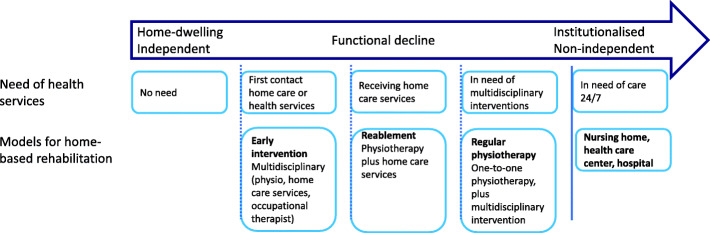
Overview of the three different models for home-based rehabilitation in Trondheim municipality in relation to the functional level and need for health services in home-dwelling older adults

Home-based physiotherapy services in Trondheim Municipality include three working models: “Regular physiotherapy”, “rehabilitation of activities of daily living” (hereafter called “reablement”), and “early intervention” (Fig. 1). These working models are organised to target older adults with different levels of physical function, independency in activities of daily life, and thus for different purposes within the primary health care. PTs provide regular physiotherapy in home- or institutional settings, after referrals from home-based services, health and welfare centres, hospitals, rehabilitation centres, general practitioners, occupational services, or by proxy. The regular physiotherapy is part of a more comprehensive, person-centred, and multidisciplinary intervention that is targeted towards the home-dwelling older adults that are the least independent in activities of daily life or have a complex health situation. The median number of home visits by the PTs in the regular physiotherapy group was 12 (interquartile range (IQR): 6–20). Reablement and early intervention consist of strategies to prevent functional decline and promote independence in activities of daily life. The decision to initiate reablement or early intervention for a patient is based on multiple factors and decided by a multidisciplinary group of health care personnel consisting of a PT, home care personnel, and an occupational therapist. Patients already receiving home-based services can be referred to physiotherapy and receive reablement, where the PTs prescribe individualised exercise programs to patients with specific focus on activities of daily living, which are followed by the home-based services. The PTs participated in three home visits as part of a multidisciplinary group, and for some patients the PTs had one to two extra home visits where they guided the patient and the home-based service in the home exercise program. Patients who contact the health services for the first time and who are not currently receiving any other health care services may receive early intervention. The patients are typically independent in activities of daily living, but need walking aids, meal delivery services, cleaning services, and/or home care alarms. Early intervention is offered to postpone the patients’ need for home-based services by education and empowerment to maintain function in activities of daily living. In this study, early intervention consists of multidisciplinary rehabilitation teams, consisting of PTs, health and welfare officers, and occupational therapists. These health care personnel were specifically trained in the early intervention model. The training consisted of workshops with a focus on assessing and uncovering early signs of functional decline, how to perform specific tests such as SPPB, and how to use motivational interviewing as a method in the rehabilitation of older adults in risk of functional decline. The health care personnel also participated in regular meetings to secure the quality of the early intervention model. The early intervention was person-centred and thus individually tailored to the patient’s goals, context, and functional status. The median number of home visits by the PTs in the early intervention group was 5 (IQR: 3–6).The early intervention model was a pilot project in the municipality that aimed to develop a new strategy for early prevention of functional decline in older adults.

Data was prospectively collected from May 2016 to May 2018. The study was conducted according to the Helsinki Declaration [12]. Written informed consent was obtained from all patients. Ethical approval was granted by the Regional committees for Medical and Health Research Ethics in Norway (REC no. 2013/2030).

### Participants

**Fig. 2:**
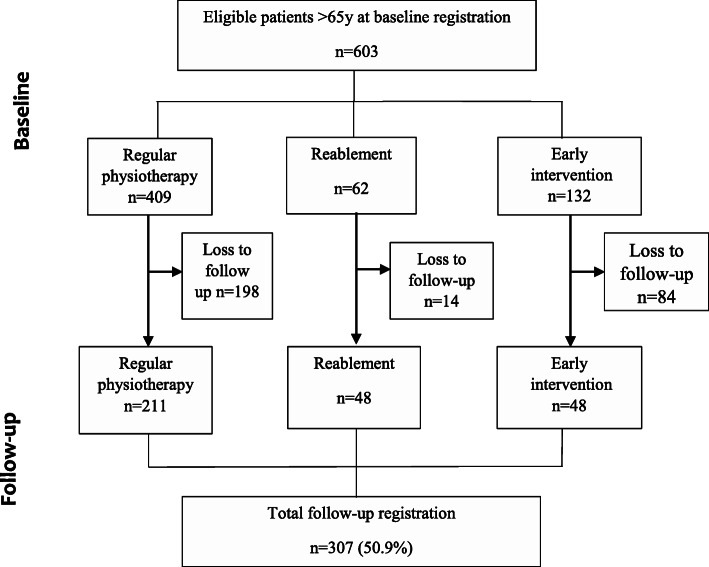
Study flow chart

Older adults ≥ 65 years, referred to home-based physiotherapy in Trondheim Municipality, Norway, were eligible for inclusion. If the participants were not able to give consent due to reduced cognitive function, the next of kin provided consent on behalf of the participant. Exclusion criteria were lack of consent in cases where the next of kin could not be reached. At baseline, 603 eligible patients had their demographic and clinical information registered, of whom 409 (67.8 %) patients received regular physiotherapy, 62 (10.2 %) received reablement, and 132 (21.9 %) received early intervention (Fig. 2).

### Data collection procedure

The PTs assessed patients at the initial home visit and used a tablet application for data collection. Data collection included self-report questionnaires and clinical tests at baseline and follow-up, defined as end of physiotherapy follow-up or maximum 6 months after baseline [11]. The median time between baseline and follow-up assessment was 94 days (IQR: 58–152) for the regular physiotherapy group, 50 days (IQR: 40–64) for the reablement group, and 112 days (IQR: 31–160) for the early intervention group.

### Assessments

At baseline, sex, age, living condition and cause of referral were registered for all patients. Physical performance, physical function, and health-related quality of life were assessed at baseline and follow-up.

Physical performance was assessed using the Short Physical Performance Battery (SPPB) [13]. The SPPB consists of three tests: standing balance, 4-meter walking at preferred speed, and five times sit-to-stand. Each test is scored on a scale from 0 to 4, which gives a composite score from 0 to 12, where lower scores indicate greater functional impairment [13]. Minimal clinically important change (MCIC) of the SPPB is defined as ≥ 1 point [14].

Physical function was assessed by using the Patient Specific Functional Scale (PSFS), which is validated for assessing changes in function for older adults [15]. The patients were asked to identify up to three important activities, which they perceived as difficult to perform due to their condition. The patients rated each activity on a scale from 0 (inability to perform) to 10 (no problem to perform). MCIC of the PSFS in older adults is lacking, although a minimal detectable change is defined as 2.8 points [15]. We used this together with results for MCIC from other patient groups [16] to define MCIC on the PSFS as ≥ 3 points. In the present study, the first activity indicated by the patient was used to assess change over time.

Health-related quality of life was assessed by using the EuroQol-5D-5 L (EQ-5D) [17], which comprises health-related quality of life in five dimensions: mobility, self-care, usual activities, pain and discomfort, and anxiety and depression. Each of these dimensions have a five-level response option; no problems, slight problems, moderate problems, severe problems and extreme problems. We reported the responses on each of the five dimensions in the EQ-5D. Further, we transformed the answers on the EQ-5D into an index score by using the English population value set [18]. The index score ranges between − 0.285 (worst imaginable health state) and 1 (perfect health). MCIC of the EQ-5D index score is defined as ≥ 0.08 points [19].

### Statistical analyses

Descriptive statistics were used to describe the patients using parametric or non-parametric statistics according to the data distribution. Mean baseline group differences in SPPB, PSFS and EQ-5D index score were analysed by one-way ANOVA with Scheffe’s post-hoc test. For the five separate dimensions in the EQ-5D we used the Kruskal Wallis test to assess the difference in baseline scores between the three different working models. Complete case analyses of SPPB, PSFS and EQ-5D index scores at baseline and follow-up were performed using paired sample t-test and scores presented as mean difference with 95 % confidence intervals (CI) for the total patient group, and for each working model separately; regular physiotherapy, reablement and early intervention. We used the Wilcoxon signed rank test to evaluate the change from baseline to follow-up in the five dimensions on the EQ-5D.

Statistical analyses were performed in STATA 15.1 (StataCorp. 2017. Stata Statistical Software: Release 15. College Station, TX: StataCorp LLC). *P*-values less than 0.05 were considered statistically significant.

## Results

### Characteristics of the three models

A total of 603 patients were included in this study (median age 84, interquartile range, IQR: 77–88 years, 65.5 % females). The age, sex and referral information of the patients in the regular physiotherapy group (*n* = 409), the reablement group (*n* = 62), and the early intervention group (*n* = 132) are shown in Table 1. The early intervention group had a higher proportion of females (73.5 %) as compared with the regular physiotherapy (62.6 %) and the reablement group (67.2 %) (*p* = 0.03). The regular physiotherapy group had a median age of 84 (IQR 76–88) years. Within this group, older adults referred to physiotherapy because of geriatrics/ functional deterioration had a median age of 87 (IQR 82–90) years. The reablement group had a median age of 85 (IQR 80–88) years, and the early intervention group a median age of 83 (IQR 79–87) years.

**Table 1:** Demographic and referral information of older patients receiving home-based physiotherapy services in Trondheim Municipality (*n* = 495). Values are reported as numbers and proportions, unless otherwise stated

	Total *n* = 603	Regular physiotherapy *n* = 409	Reablement *n* = 62	Early intervention *n* = 132
Age				
median, IQR	84 (77–88)	84 (76–88)	85 (80–88)	83 (79–87)
65–74	108 (17.9)	87 (21.3)	6 (9.7)	15 (11.4)
75–84	212 (35.2)	122 (29.8)	24 (38.7)	66 (50.0)
>85	283 (46.9)	200 (48.9)	32 (51.6)	51 (38.6)
Sex, female	394 (65.5)	256 (62.6)	41 (67.2)	97 (73.5)
Referral from				
Patient self/relatives	145 (24.1)	72 (17.6)	-	73 (55.3)
Doctor	118 (19.6)	85 (20.8)	-	33 (25.0)
Health and welfare centre	57 (9.5)	54 (13.2)	-	3 (2.3)
Homecare services	91 (15.1)	29 (7.1)	62 (100)	1 (0.8)
Specialist health care	96 (15.9)	84 (20.5)	-	12 (9.1)
Other	95 (15.8))	85 (20.8)	-	10 (7.6)

*IQR *interquartile range, *SD *standard deviation, *NRS *Numeric Rating Scale, *SBBP *Short Physical Performance Battery, *PSFS *Patient Specific Functional Scale, *EQ-5D *EuroQol health-related quality of life questionnaire

The most frequent causes of referral to regular physiotherapy were geriatrics/functional deterioration with or without falls (*n* = 166, 40.6 %), orthopaedic (*n* = 79, 19.3 %) and neurological conditions (*n* = 45, 11 %), musculoskeletal disorders (*n* = 53, 13.0 %), and other causes (*n* = 66, 16.1 %) including cancer, heart and pulmonary diagnoses, and congenital diagnoses. In all groups, physiotherapy treatment consisted mostly of gait training, activities of daily living, and stair walking. The most frequently used exercises were strengthening exercises and balance/stability/coordination exercises.

Table 2 shows the baseline SPPB, PSFS and EQ-5D scores. At baseline, the early intervention group had significantly higher SPPB and EQ-5D index scores than the regular physiotherapy and reablement groups (Scheffe’s *p* value *p* < 0.05). For the PSFS score the largest difference were observed between the early intervention and the regular physiotherapy group. The EQ-5D dimensions mobility, personal care, and usual activities at baseline showed statistically significant differences between the three working models. The regular physiotherapy group had lower scores on the mobility and personal care dimension compared to the reablement and early intervention group. The early intervention group showed higher scores on the usual activities dimension compared to the reablement and regular physiotherapy group. The EQ-5D dimensions pain/discomfort and anxiety/depression were not significantly different between the three working models at baseline. For the pain/discomfort dimension the proportion reporting moderate to severe problems was 50.1 %, 39.8 %, and 44.8 % in the regular physiotherapy group, reablement group and the early intervention group, respectively.

**Table 2:** Baseline scores and within group changes from baseline to post-treatment assessment in physical performance, physical function, and health-related quality of life dimensions in adult and older patients receiving home-based physiotherapy services in Trondheim Municipality

	Total sample (*n* = 603)	*n*	Regular physiotherapy (*n* = 409)	*n*	Reablement (*n* = 132)	*n*	Early Intervention (*n* = 62)	*n*	Between group difference *p*-value^1^
**Short Performance Physical Battery** (0–12, higher score indicate better performance)									
Baseline, mean (SD)	6.3 (3.0)	373	5.6 (2.8)	200	5.5 (2.6)	113	7.9 (2.7)	60	< 0.001
Post-treatment, mean (SD)	7.1 (2.7)	130	7.2 (2.5)	66	6.6 (2.7)	43	8.5 (2.8)	19	0.04
Change, mean (95 % CI)	0.9 (0.6 to 1.1)	128	1.0 (0.7 to 1.4)	66	0.8 (0.4 to 1.3)	43	0.4 (-0.2 to 1.0)	19	0.25
**Patient Specific Functional Scale** (0–10, higher score indicate better function)									
Baseline, mean (SD)	3.6 (2.8)	431	3.3 (2.9)	298	4.1 (2.5)	90	4.6 (2.4)	43	< 0.001
Post-treatment, mean (SD)	6.1 (3.2)	217	6.1(3.3)	153	5.8 (3.2)	33	6.4 (2.7)	31	0.75
Change, mean (95 % CI)	2.3 (1.8 to 2.8)	217	2.5 (1.9 to 3.2)	153	1.8 (0.5 to 3.1)	33	1.7 (0.8 to 2.6)	31	0.34
**Health related Quality of Life, EQ-5D** (0–1, higher score indicate better quality of life									
Baseline, mean (SD)	0.46 (0.22)	551	0.41(0.22)	383	0.50 (0.15)	110	0.59 (0.19)	58	< 0.001
Post-treatment, mean (SD)	0.56 (0.21)	380	0.54 (0.23)	190	0.60 (0.16)	45	0.61 (0.17)	45	0.04
Change, mean (95 % CI)	0.10 (0.07 to 0.12)	268	0.11 (0.08 to 0.14)	183	0.10 (0.06 to 0.14)	43	0.04 (-0.01 to 0.09)	42	0.11
EQ-5D Mobility									
Baseline, median (IQR)	3 (2–3)	554	3 (2–4)	388	2 (2–3)	58	2 (2-2.5)	108	< 0.001
Post-treatment, median (IQR)	2 (1–3	286	2 (1–3)*	197	2 (1–3)*	45	2 (1–2)	44	0.13
EQ-5D Personal care									
Baseline, median (IQR)	2 (1–2)	553	2 (1–3)	387	1 (1–2)	58	1 (1–1)	108	< 0.001
Post-treatment, median (IQR)	1 (1–2)	290	1 (1–2)*	197	1 (1–2)*	45	1 (1-1.5)	48	0.002
EQ-5D Usual activities									
Baseline, median (IQR)	3 (2–4)	552	3 (2–4)	386	2.5 (2–3)	58	2 (1–2)	108	< 0.001
Post-treatment, median (IQR)	2 (1–3)	289	2 (2–3)*	196	2 (1–2)*	45	2 (1–2)	48	< 0.001
EQ-5D Pain/discomfort									
Baseline, median (IQR)	2 (2–3)	554	3 (2–3)	388	2 (2–3)	58	2 (1.5-3)	108	0.08
Post-treatment, median (IQR)	2 (1–3)	285	2 (1–3)*	195	2 (1-2.5)*	44	2 (2–3)	46	0.68
EQ-5D Anxiety/depression									
Baseline, median (IQR)	2 (1–2)	552	2 (1–2)	386	2 (1–2)	58	1 (1–2)	108	0.42
Post-treatment, median (IQR)	1 (1–2)	283	1 (1–2)^*^	193	1.5 (1–2)	44	1 (1–2)	46	0.69

*CI *confidence interval, *EQ-5D *EuroQol health-related quality of life questionnaire

^1^*p*-value from analysis of variance or chi-square test

^*^p-value < 0.05; Wilcoxon signed rank test

### Change from baseline to follow-up

In total 402 out of the 603 patients completed both baseline and follow-up assessments. Table 2 shows the follow-up overall and within group mean changes (95 % CI) in SPPB, PSFS and EQ-5D scores (complete cases). Overall, the mean SPPB scores improved by 0.9 (95 % CI: 0.6 to 1.1) points from baseline to follow-up, mean PSFS scores improved by 2.3 (95 % CI: 1.8 to 2.8) points, and mean EQ-5D scores improved by 0.10 (95 % CI: 0.07 to 0.12) points. The regular physiotherapy and reablement groups showed significant improvements in SPPB, PSFS and EQ-5D scores, while the early intervention group only showed significant improvement in PSFS scores (mean change:1.7; 95 % CI: 0.8 to 2.6). Proportions of patients with clinically relevant changes in SPPB, PSFS, and EQ-5D in the regular physiotherapy group were 36 %, 44 %, and 52 %, respectively. In the reablement group it was 56 %, 30 %, and 58 %, and in the early intervention group it was 36 %, 35 %, and 38 %, respectively.

### Missing data

There were no significant differences in baseline demographic and clinical characteristics between complete cases and patients missing at follow-up. One exception was higher baseline SPPB scores for the complete cases in the regular physiotherapy as compared to patients missing at follow-up (regular physiotherapy: 6.1 (SD 2.5) vs. 5.2 (SD 2.9), *p* = 0.02).

## Discussion

The present study is the first to describe characteristics and outcomes of patients in different organisational models of home-based rehabilitation in a primary health care setting in Norway. The patients were heterogeneous in terms of degree of functional deterioration and in health-related quality of life across the different models of home-based rehabilitation, although minor differences were observed between those receiving reablement and regular physiotherapy. The early intervention group had better physical performance and health-related quality of life compared with the other two groups. At follow-up, the regular physiotherapy group and the reablement group improved their physical performance, physical function, and health-related quality of life, while the early intervention group improved in physical function.

### Demographic and clinical characteristics of patients receiving home-based rehabilitation

Patients receiving regular physiotherapy with geriatrics/functional deterioration as cause of referral were only slightly older than those receiving reablement and early intervention. With a median age of 86.5 years, these patients represent an older patient group compared with other studies of home-dwelling older adults [3, 20–24], and similar age to those admitted to nursing homes [10]. The patients receiving reablement in the current study are older compared to patients receiving reablement in other areas of Norway [3], as well as in other western countries [4, 9]. The main reason for the age difference can be the inclusion criteria of age > 65 years used in the present study, whereas other studies have included subjects below 65 years [3, 4]. Further, the longer life expectancy in Norway (83 years) compared to 81 years in UK and 79 years in US could also have influenced the age differences between the studies [25].

The mobility level in the reablement and the regular physiotherapy groups was similar and considerably lower than in the early intervention group, and similar to other studies investigating older adults receiving reablement or usual care in primary health care [3, 9]. In the present study, the regular physiotherapy group had poorer health-related quality of life compared to the reablement group, which indicates that older adults receiving regular physiotherapy have a more complex health situation. This is in line with the differentiation in working models made by the municipality for patients receiving reablement versus the regular physiotherapy group, where the latter receive more comprehensive and multidisciplinary interventions in addition to the home-based regular physiotherapy. A study investigating users of reablement services in the UK found similar health related quality of life as the reablement group in the current study, but unfortunately, the UK study did not perform objective measurement of mobility [4]. This suggests that although patients receiving home-based rehabilitation in the current study are older than in other areas in Norway and other countries, they have the same mobility level and health related quality of life.

### Changes from baseline to follow-up

The reablement and the regular physiotherapy group had comparable changes in mobility and health-related quality of life, and in proportion of patients achieving clinically meaningful improvement on these outcomes. The early intervention group had only minor changes in these outcomes. These results reflect the different aims and target groups of the working models, where the early intervention was targeted at prevention of functional decline and that the higher baseline values for physical performance and health-related quality of life in early intervention may have reduced the potential to achieve large improvements. However, the early intervention group showed similar changes in PSFS scores as the other groups. In the current study, the PTs and the patients collaborated to identify the most important activities at the start of treatment, and they evaluated the progress after the treatment. The strength of using PSFS as an outcome measure and to monitor treatment progress at an individual level is that the PSFS is person-centred, in contrast to SPPB and EQ-5D where activities and questions are pre-specified and generic for all patients [26]. Therefore, the use of PSFS in treatment planning and monitoring complements the use of other traditional generic or condition-specific questionnaires, and have the potential to capture relevant changes in older adults where the interventions are aimed at prevention and proactive health services, such as in the early intervention group.

### Strengths and limitations

Strengths of the present study were the systematic collection of data of patients receiving home-based physiotherapy services, the inclusion of self-reported and objective measures of physical performance before and after physiotherapy. A limitation of this study was that not all patients referred to home-based physiotherapy included. The PTs reported that reasons for exclusion were reduced cognitive function, incapable of giving consent or the PT not being able to reach the next of kin, language barriers for non-Norwegian speaking patients and ethical concerns for patients receiving palliative care. We have previously reported that sex and age distribution of adult and older patients included in FYSIOPRIM were comparable to those not included, although a higher proportion of non-included patients were referred because of geriatrics/functional deterioration [11]. Our sample may therefore have had higher physical performance compared with all patients receiving home-based physiotherapy. The lower number of patients in the early intervention group may have reduced the power to detect baseline differences between the working models and changes from baseline to follow-up. However, we found baseline differences between the groups and the non-significant changes from baseline to follow-up in the early intervention group were small and clinically not relevant according to the MCIC defined in the current study. There was a considerable amount of missing data at follow-up, which reflect the challenges observed in other studies with obtaining follow-up data from older patients in home-based rehabilitation settings, where an attrition rate of 50 % was observed [4]. We did not find any baseline differences in demographic and clinical characteristics between those with and without complete follow-up data, except complete cases in the regular physiotherapy group had higher SPPB scores, and thus better physical performance, than patients missing data at follow-up. However, this did not influence the fact that we found a significant improvement from baseline to follow-up in SPPB. As the present study relied on assessments by up to 55 different PTs during clinical encounters with the patients [11], inter-rater reliability bias may have occurred, as well as potential desirability bias as all patient-reported outcome measures were registered with the PT present. As the SPPB is an integrated part of the physiotherapy practice and most PTs are experienced users of the instrument, we expect the inter-rater reliability for this measure to be acceptable.

### Implications for health services

Identification of older adults at risk for developing long-term disability is important to secure an independent life by postponing the need for health care services, admission to nursing homes, and hospitalisation. A recent study from Norway investigating mobility level and performance in 697 older adults at admission to nursing homes reported a median SPPB score of 4 points [10]. The difference in mobility level between home-dwelling older adults in the current study and older adults admitted to nursing homes are therefore surprisingly small. Interventions such as reablement and early intervention, where patients are at a higher mobility level, have the potential to initiate physiotherapy at an earlier stage and potentially postponing admissions to nursing homes. Identification of older adults at risk for functional decline, and thus a potential candidate for reablement or early intervention, was done by a multidisciplinary group that was trained in detecting early signs of functional decline. Therefore, the multidisciplinary approach used to allocate patients to the most appropriate intervention, together with the multidisciplinary treatment approach in the early intervention model are a promising organisational model for home-based rehabilitation in primary care.

The early intervention model was initiated as the municipality experienced that home-based rehabilitation started too late, when the patients already had a substantially reduced functional level with limited potential for prevention of functional decline. By use of a model for early intervention, as shown in a former study, patients were identified at an earlier stage as indicated by the SPPB [3]. In line with this, we also showed that we identified persons at an earlier stage, but their mobility levels were already starting to decline when they sought home-based services for the first time. Thus, physiotherapy or other preventive interventions could be initiated at an even earlier stage to decrease the risk for long-term mobility-related disability. The present study suggests that the specific training of multidisciplinary health care personnel in combination with a well-organised health care system within the municipality is important to develop a targeted prevention and rehabilitation strategy, as shown in the early intervention model in the present study.

## Conclusions

Older adults receiving reablement or regular physiotherapy had similar patient characteristics, and showed similar improvements in physical function, physical performance and health-related quality of life. Early intervention identified older patients at risk of functional decline at an earlier stage compared to reablement and regular physiotherapy. These findings have implications for future research and for policy makers in the health services as we show that different organisational models of home-based rehabilitation may influence the initiation of proactive health services and the potential for early prevention of functional decline in older patients at risk of functional decline and reduced independence in daily life. These findings can thus be used for designing and improving rehabilitation strategies within primary health care.

## Data Availability

The datasets generated and/or analysed during the current study are not publicly available due to permission has not been applied for from neither the participants nor the Ethical Committee, but might be available from the corresponding author on reasonable request.

## Abbreviations

CI: Confidence interval
EQ-5D: EuroQol, five dimensions
FYSIOPRIM: Physiotherapy in the primary health care
IQR: Interquartile range
MCIC: Minimum clinically important change
PSFS: Patient specific functional scale
SD: Standard deviation
SPPB: Short physical performance battery
